# A CT-based diagnostic nomogram and survival analysis for differentiating grade 3 pancreatic neuroendocrine tumors from pancreatic ductal adenocarcinomas

**DOI:** 10.3389/fonc.2024.1443213

**Published:** 2024-08-29

**Authors:** Miaomiao Hu, Lulu Lv, Hongfeng Dong

**Affiliations:** ^1^ Department of Radiology, The First People’s Hospital of Huzhou, Huzhou, China; ^2^ Department of Radiology, Xuzhou Central Hospital, Xuzhou, China

**Keywords:** pancreatic neuroendocrine tumour, neuroendocrine carcinoma, pancreatic ductal adenocarcinoma, nomogram, computed tomography

## Abstract

**Objective:**

To construct a CT-based diagnostic nomogram for distinguishing grade 3 pancreatic neuroendocrine tumors (G3 PNETs) from pancreatic ductal adenocarcinomas (PDACs) and assess their respective survival outcomes.

**Methods:**

Patients diagnosed with G3 PNETs (n = 30) and PDACs (n = 78) through surgery or biopsy from two medical centers were retrospectively identified. Demographic and radiological information, including age, gender, tumor diameter, shape, margin, dilatation of pancreatic duct, and invasive behavior, were carefully collected. A nomogram was established after univariate and multivariate logistic regression analyses. The Kaplan–Meier survival was performed to analyze their survival outcomes.

**Results:**

Factors with a p-value <0.05, including age, CA 19-9, pancreatic duct dilatation, irregular shape, ill-defined margin, pancreatic atrophy, combined pancreatitis, arterial/portal enhancement ratio, were included in the multivariate logistic analysis. The independent predictive factors, including age (OR, 0.91; 95% CI, 0.85–0.98), pancreatic duct dilatation (OR, 0.064; 95% CI, 0.01–0.32), and portal enhancement ratio (OR, 1,178.08; 95% CI, 5.96–232,681.2) were determined to develop a nomogram. The internal calibration curve and decision curve analysis demonstrate that the nomogram exhibits good consistency and discriminative capacity in distinguishing G3 PNETs from PDACs. Patients diagnosed with G3 PNETs exhibited considerably better overall survival outcomes compared to those diagnosed with PDACs (median survival months, 42 vs. 9 months, p < 0.001).

**Conclusions:**

The nomogram model based on age, pancreatic duct dilatation, and portal enhancement ratio demonstrates good accuracy and discriminative ability effectively predicting the probability of G3 PNETs from PDACs. Furthermore, patients with G3 PNETs exhibit better prognosis than PDACs.

## Introduction

Pancreatic neuroendocrine tumors (PNETs) are rare and heterogeneous neoplasms arising in the islets of Langerhans (1). The incidence of PNETs has continued to steadily increase in the last three decades, particularly in older people possibly thanks to stage migration and detection of early-stage disease (2, 3). According to the 2022 World Health Organization classification (4), all PNETs are regarded as malignant tumors and could be stratified into grade 1 (G1), grade 2 (G2), and grade 3 (G3), as G3 was further divided into well-differentiated neuroendocrine tumors (NETs) and poorly differentiated NETs, also named as pancreatic neuroendocrine carcinomas (PNECs). G3 PNETs were frequently misdiagnosed as pancreatic ductal adenocarcinomas (PDACs) due to shared radiologic characteristics, including invasive appearance, relatively poor vascular enhancement, ill-defined margins, and dilation of the pancreatic duct (5, 6).

The 5-year overall survival rate differed significantly by grade, stage, primary site, age at diagnosis, and time period of diagnosis; even patients with metastases have a median survival time of 2 years (2). PNETs are indolent resulting in much better long-term survival outcomes when compared with PDACs (7). The median survival time of G3 PNETs was 36 months when compared to PDACs, which was only 8 months (5). The portal enhancement ratio <1.02 and lymph node metastases were independent prognostic variables for worse outcomes in PNETs as a study showed (8).

However, due to the very rare G3 tumors accounting for only 2%–3% of all PNETs (9, 10), we know little about it. Moreover, many radiologic studies related to PNETs were mostly G1/G2 or focused on non-hypervascular, non-functioning PNETs (6, 11–13). The European neuroendocrine tumor society recommended that the nomogram after PNET resection may contribute to estimating the risk of recurrence and help to schedule the clinical follow-up indicating the importance of the nomogram (14). Therefore, our study aimed to establish a CT-based diagnostic nomogram for distinguishing G3 PNETs from PDACs and compare their survival outcomes.

## Materials and methods

### Patients

The institutional review board approved this retrospective study, and the requirement for informed consent was waived.

Patients with pathologically proven G3 PNETs (containing 15 cases of PNECs) at two hospitals from 1 January 2010, up to 1 March 2024, and patients with PDACs from 1 January 2018, up to 1 January 2024, were retrospectively analyzed.

The inclusion criteria were as follows: 1) diagnosis as G3 PNETs or PDACs confirmed by surgery resection or liver metastasis/lymph nodes/primary biopsy, 2) preoperative contrast-enhanced CT within 30 days before surgery or biopsy, and 3) no local treatment or chemotherapy before operations. The exclusion criteria were as follows: 1) patient with severe pancreatic atrophy resulting in lack of region of interest when measuring CT Hounsfield unit (HU), and 2) no available images or poor images. Finally, we included 30 patients with G3 PNETs and 78 PDACs in our study.

### Imaging acquisition

All patients were asked to fast from solid form for 4–6 h before the examination. CT images were performed using Siemens Somatom Definition 128 AS (Siemens Medical Systems) and GE APEX 256 CT (GE Healthcare). First, patients underwent plain CT scans, and then a non-ionic contrast medium (Omnipaque 300 g/L, GE Healthcare) was intravenously injected at a rate of 2.5 ml/s. Arterial phase and portal venous phase were attained, respectively. CT images were contained at 120 kVp and 200 mAs, with beam pitch of 0.984, slice collimation of 0.75 mm, slice thickness of 3–5 mm, and gantry rotation time of 0.5 s. The imaging delay time for arterial and portal venous phases was 18–19 and 40–50 s, respectively.

### Imaging analysis

All images were analyzed by two abdominal radiologists (10 and 7 years of experience in abdominal radiology, respectively), who were unaware of the pathology. Disagreements in assessment between them were settled by consensus after consultation with a third radiologist, who has a 23 years of clinical experience in pancreatic radiology.

Demographic information [age, gender, symptoms, carbohydrate antigen (CA) 19-9, overall survival outcome] and general radiological information (location, tumor maximum diameter, pancreatic atrophy, combined pancreatitis) were carefully collected. Whereafter, they also evaluated the tumor shape, texture, tumor margin, dilatation of pancreatic main duct, lymph node enlargements, liver metastases, and invasion of surrounding tissues. Symptoms include abdominal pain, jaundice, abdominal distension, discomfort, and weight loss. Overall survival was determined from the date of biopsy or surgery to the date of death. Patients who remained alive at the last observed follow-up date (1 March 2024) or those who were lost to follow-up without providing a reason were censored in the analysis.

Tumor shape was divided into round and irregular shapes. Tumor margin was classified as well defined and ill defined. A well-defined margin was considered a smooth contour without speculation or infiltration in more than 80% of the perimeter of the tumor, while an ill-defined margin was with speculation or infiltration in more than 20% of the perimeter of the tumor (15). Tumour texture was categorized as solid and combined with cystic types. The solid type was suggested as an enhancing solid component of more than 90% of the whole tumor combined with the cystic type as an enhancing solid component of less than 90% of the whole tumor (6). Pancreatic duct dilatation was defined when its main duct diameter was ≥ 3 mm (6). Lymph node enlargement was regarded when its short-axis diameter was >5 mm or contained necrosis in any size (9). Liver metastases were considered as multiple peripheral enhanced or hypervascularity nodules (16). Invasion of nearby tissues was defined as tumor invading adjacent organs/tissues or large vessels. On CT plain images, plain ratio was considered as HU of the lesion/HU of the adjacent pancreas. As for CT-enhanced images, arterial enhancement ratio, defined as the arterial HU of the tumor/the same phase of HU of the adjacent pancreas, and portal enhancement ratio, also defined in the same way but in portal phase, were carefully calculated (16).

### Statistical analysis

Qualitative data were presented as frequencies and percentages, while quantitative data were expressed as mean ± standard deviation or as median (25th–75th percentile) depending on the distribution. Differences in continuous variables between subgroups were assessed using the Student’s t-test or the Mann–Whitney U test. Qualitative variables were compared using Fisher’s exact test or the χ2 test. Multivariable binary regression analysis was conducted to identify independent factors distinguishing G3 PNETs from PDACs, with odds ratios (OR) and 95% confidence interval (CI) calculated. A nomogram for differentiating G3 PNETs from PDACs was developed based on independent predictive factors confirmed by multivariable logistic analysis. The calibration curve and decision curve of the nomogram were validated to assess its discriminative ability in distinguishing G3 PNETs from PDACs, respectively. The nomogram was constructed using R 4.3.2 software (http://www.r-project.org/). Kaplan–Meier survival analysis was performed to generate survival curves, and the log-rank test was used to compare overall survival outcomes between G3 PNET and PDAC groups using Stata software (version 16.0, StataCorp). Additional statistical analysis was conducted using SPSS (version 23.0, IBM company). All tests were two sided, and statistical significance was considered at a confidence level of 0.05.

## Results

### Baseline demographic and radiologic data of the patients

The clinical and imaging information is summarized in **Table 1**. Our study included 30 patients diagnosed with G3 PNETs and 78 patients diagnosed with PDACs. In the G3 PNET group, we found that among the 15 cases of PNECs, 11 had invasion of nearby tissues, 7 had liver metastases, and 5 had lymph node metastases. In contrast, among the 15 cases of PNETs, 9 showed invasion of surrounding tissues, only 1 had liver metastasis, and none had lymph node metastasis. Among these cases, PDAC patients were more frequently older (median age, 64.0 vs. 58.0, p < 0.05) and exhibited abnormal levels of CA 19-9 (85.9% vs. 53.3%) compared to G3 PNET patients. However, no significant differences were observed in terms of gender and symptoms between the two groups. PDACs were more likely to present with pancreatic duct dilatation (62.8% vs. 20.0%), irregular shape (97.4% vs. 70.0%), ill-defined margin (98.7% vs. 66.7%), pancreatic atrophy (39.7% vs. 13.3%), and pancreatitis (47.4% vs. 23.3%), compared with G3 PNETs (all p < 0.05). Although the size of G3 PNETs tended to be larger than that of PDACs (median diameter, 3.8 vs. 3.2 cm), the difference did not reach statistical significance. Additionally, more cystic components (43.3% vs. 37.2%) and liver metastases (30.0% vs. 19.2%) were observed in G3 PNETs compared to PDACs, with no significant difference. Furthermore, no significant differences were found in terms of lesion location, invasion of nearby tissues, and lymph node enlargement between G3 PNETs and PDACs.

**Table 1:** Baseline demographic and radiologic characteristics of the study patients.

	All subjects (n = 108)	G3 (n = 30)	PDAC (n = 78)	p-Value
Age	62.5 (54, 66)	58.0 (49.5, 64.3)	64.0 (58.8, 67.3)	**0.009**
Gender				0.362
Male	72 (66.7)	18 (60.0)	54 (69.2)	
Female	36 (33.3)	12 (40.0)	24 (30.8)	
Symptoms	90 (83.3)	26 (86.7)	64 (82.1)	0.774
CA 19-9	83 (76.9)	16 (53.3)	67 (85.9)	**<0.001**
Location				0.595
Head	62(57.4)	16 (53.3)	46 (59.0)	
Body/tail	46 (42.6)	14 (46.7)	32 (41.0)	
Largest diameter (cm)	3.6 (2.4, 5.5)	3.8 (2.7, 5.5)	3.2 (2.3, 5.6)	0.312
Pancreatic duct dilatation	55 (50.9)	6 (20.0)	49 (62.8)	**<0.001**
Irregular shape	97 (89.8)	21 (70.0)	76 (97.4)	**<0.001**
Ill-defined margin	97 (89.8)	20 (66.7)	77 (98.7)	**<0.001**
Tumor texture				0.557
Solid	66 (61.1)	17 (56.7)	49 (62.8)	
Combined with cystic	42 (38.9)	13 (43.3)	29 (37.2)	
Pancreatic atrophy	35 (32.4)	4 (13.3)	31 (39.7)	**0.011**
Combined pancreatitis	44 (40.7)	7 (23.3)	37 (47.4)	**0.022**
Invasion of nearby tissues	73 (67.6)	20 (66.7)	53 (67.0)	0.899
Lymph nodes enlargement	28 (25.9)	5 (16.7)	23 (29.5)	0.224
Liver metastases	24 (22.2)	9 (30.0)	15 (19.2)	0.228
Plain ratio	0.91 ± 0.23	0.99 ± 0.27	0.89 ± 0.21	0.083
Arterial enhancement ratio	0.68 ± 0.23	0.79 ± 0.29	0.64 ± 0.19	**0.013**
Portal enhancement ratio	0.71 ± 0.19	0.83 ± 0.18	0.67 ± 0.17	**<0.001**

The bold p-values indicate p < 0.05, which is statistically significant.

Regarding quantitative CT HU values, no significant difference was observed in the plain ratio between G3 PNETs and PDACs (0.99 ± 0.27 vs. 0.89 ± 0.21, p > 0.05). G3 PNETs exhibited higher vascular enhancement both in the arterial (0.79 ± 0.29 vs. 0.64 ± 0.19) and portal venous (0.83 ± 0.18 vs. 0.67 ± 0.17) phases compared to PDACs. Both lesions appeared hypo-enhancing relative to the normal pancreatic parenchyma on arterial and portal venous phases and demonstrated progressive enhancement, especially in G3 PNETs.

### Logistic regression analysis for tumor differentiation

Based on the previous univariate analysis, factors with a p-value <0.05 were included in the subsequent multivariate binary logistic regression analysis. These factors included age, CA 19-9, pancreatic duct dilatation, irregular shape, ill-defined margin, pancreatic atrophy, combined pancreatitis, arterial enhancement ratio, and portal enhancement ratio (as shown in **Table 2**). Following the logistic analysis, three factors—age, pancreatic duct dilatation, and portal enhancement ratio—were identified as independent predictive factors for distinguishing G3 PNETs from PDACs. Specifically, younger age (OR, 0.91; 95% CI, 0.85–0.98), absence of pancreatic duct dilatation (OR, 0.064; 95% CI, 0.01–0.32), and larger portal enhancement ratio (OR, 1,178.08; 95% CI, 5.96–232,681.2) were determined as independent predictive factors for G3 PNETs.

**Table 2:** Multivariate logistic regression analysis between G3 PNETs and PDACs.

Variables	OR	95% CI	p-Value
Age (years)	0.91	0.85, 0.98	**0.011**
CA 19-9	0.76	0.20, 2.89	0.684
Pancreatic duct dilatation	0.064	0.01, 0.32	**0.001**
Irregular shape	0.49	0.03, 0.39	0.607
Ill-defined margin	0.35	0.02, 0.54	0.441
Pancreatic atrophy	0.96	0.21, 4.38	0.959
Combined pancreatitis	1.14	0.28, 4.69	0.853
Arterial enhancement ratio	0.56	0.02, 16.63	0.736
Portal enhancement ratio	1,178.08	5.96, 232,681.2	**0.009**

OR, odds ratio; CI, confident interval. The bold p-values indicate p < 0.05, which is statistically significant.

### Nomogram establishment and validation

**Figure 1:**
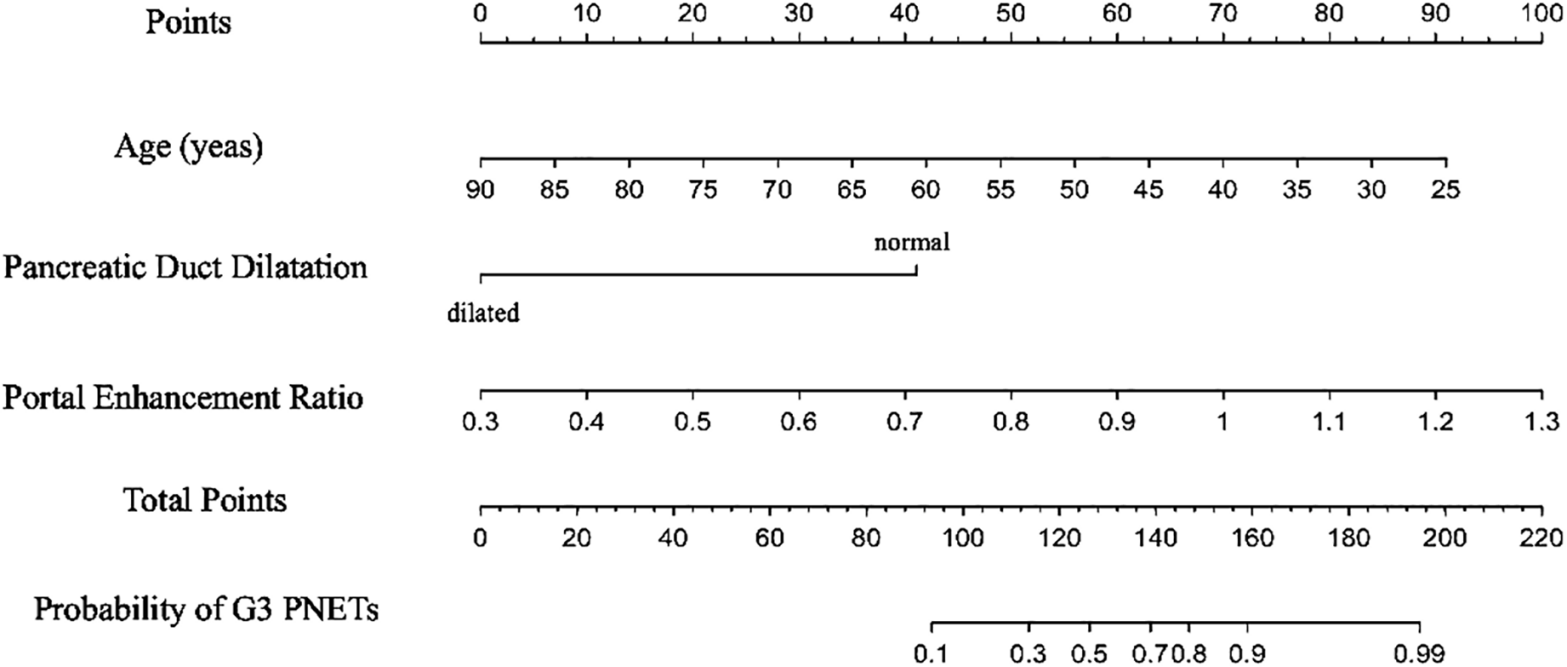
A nomogram, containing age, pancreatic duct dilatation, and portal enhancement ratio for predicting the probability of G3 PNETs, was established. Higher total scores indicate a greater likelihood of G3 PNETs compared to PDACs. Upon achieving a total score of 160 points, the probability of G3 PNET presence is up to 90%, whereas the probability is less than 10% if the total score is less than 90 points.

**Figure 2:**
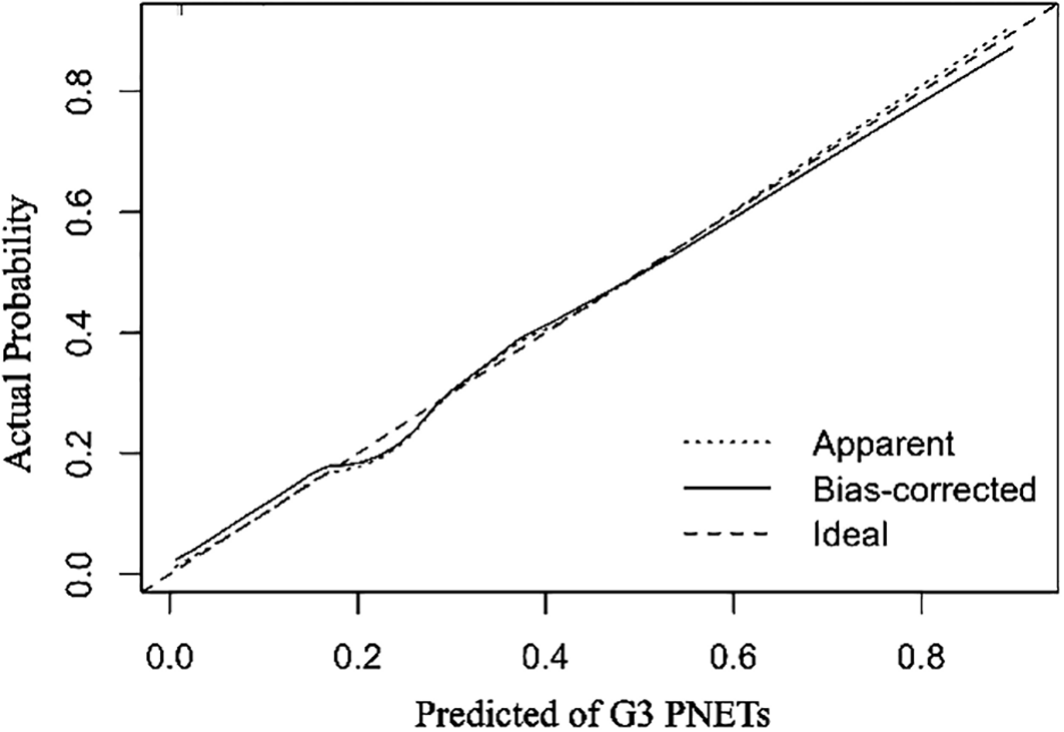
The internal calibration curves demonstrate that the nomogram exhibits good fidelity and consistency in predicting G3 PNETs probabilities.

**Figure 3:**
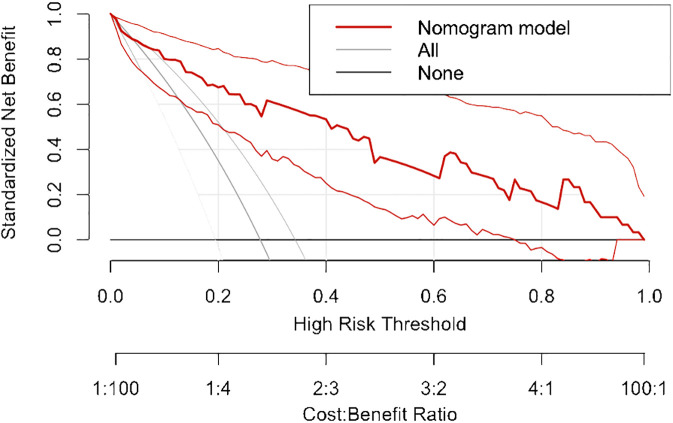
Decision curve analysis indicates that the nomogram possesses good precision and discriminative capacity in distinguishing G3 PNETs from PDACs.

All independent diagnostic variables identified in the logistic analysis were utilized to develop the nomogram (**Figure 1**). This nomogram comprises calibrated scales or axes for each predictor variable, where a straight line drawn between each predictor’s value and the probability axis denotes the contribution of that variable to the overall prediction. By aligning the values of each predictor on the scales and summing their contributions, the predicted probability of G3 PNETs can be easily determined. Higher total scores indicate a greater likelihood of G3 PNETs compared to PDACs. Upon achieving a total score of 160 points, the probability of G3 PNETs presence is up to 90%, whereas the probability is less than 10% if the total score is less than 90 points. The internal calibration curve demonstrates that the nomogram exhibits good fidelity and consistency in predicting G3 PNET probabilities (**Figure 2**). Decision curve analysis indicates that the nomogram possesses good precision and discriminative capacity in distinguishing G3 PNETs from PDACs (**Figure 3**).

### Survival outcomes of the patients

**Figure 4:**
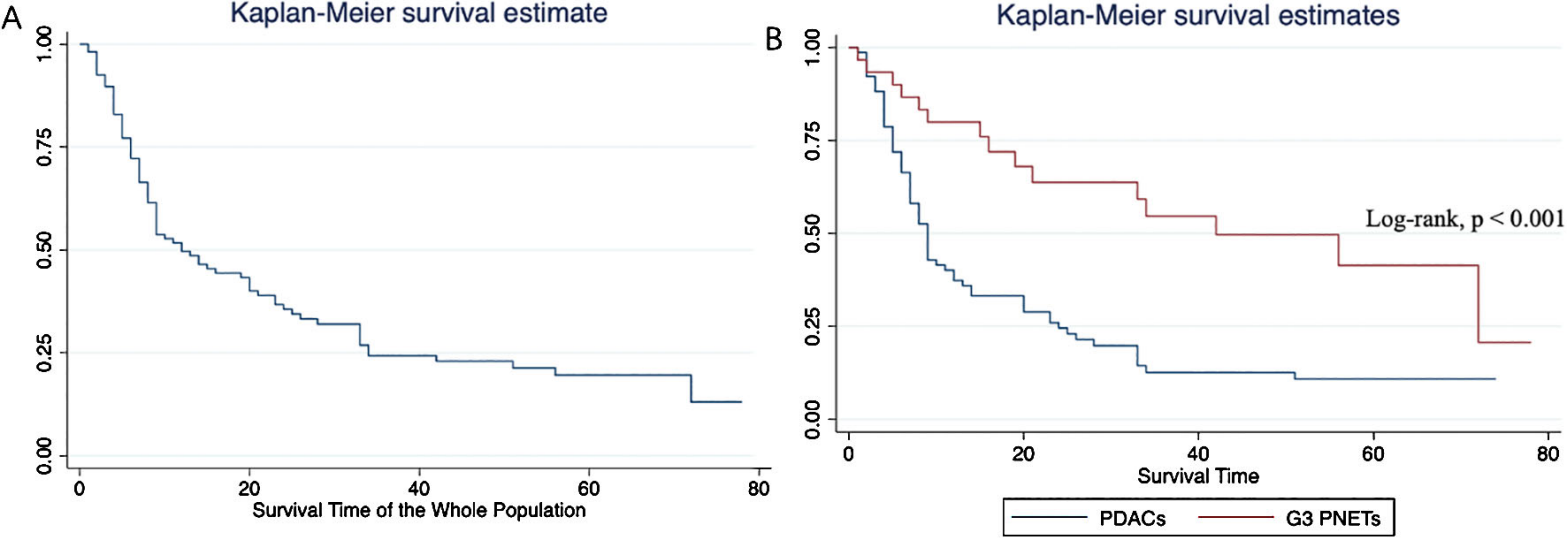
Overall survival analyses of the whole population **(A)** and subgroups **(B)**. Patients diagnosed with G3 PNETs exhibited considerably better overall survival outcomes compared to those diagnosed with PDACs.

The median overall survival of the entire population was 12 months. Specifically, the median survival of G3 PNETs was 42 months (25th–75th percentile, 16–72 months), whereas the median survival of PDACs was only 9 months (25th–75th percentile, 5–24 months). The Kaplan–Meier curve depicting the survival outcomes of all patients, as well as those specifically with G3 PNETs and PDACs, is illustrated in **Figure 4**. This graph demonstrates a significant difference in survival outcomes between the two subgroups (log-rank p < 0.001). Notably, patients diagnosed with G3 PNETs exhibited considerably better overall survival outcomes compared to those diagnosed with PDACs.

## Discussion

In our study, we identified age, pancreatic duct dilatation, and portal enhancement ratio as independent predictive factors for distinguishing between G3 PNETs and PDACs. The diagnostic nomogram developed based on these factors demonstrated good accuracy and discriminative ability effectively predicting the probability of G3 PNETs. Additionally, patients with G3 PNETs exhibited significantly longer median survival times compared to those with PDACs suggesting a better prognosis for G3 PNET patients. The nomogram established in our study may facilitate the development of more personalized treatment strategies and approaches for prognosis assessment.

Distinguishing between G3 PNETs and PDACs poses a recognized diagnostic challenge. Accurate classification is crucial as clinical behavior, survival outcomes, and treatment strategies vary between these entities. In evaluating pancreatic tumors, imaging has been a mainstay in their diagnosis and also plays a paramount role in guiding appropriate therapeutic approach (17). A hyper- or iso-enhancement in the portal venous phase and well-defined margin were more frequently shown in PNETs than in PDACs (6), which was also found in our results. The median diameter of G3 PNETs was 3.8 cm, whereas that of PDACs was 3.2 cm in our study. G3 PNETs tend to grow larger, which may be due to high cellularity, active proliferative index, and lower differentiation resulting in necrosis within the lesion, especially in PNECs (18). Pancreatic duct dilation is an independent risk factor for predicting the lymph node metastasis of PNETs (19). However, PNETs were less commonly associated with dilation of the pancreatic duct than that of PDACs (12). Chen et al. (5) found that a normal level of CA 19-9, normal pancreatic duct dilation, and round shape were predictive factors of G3 PNETs than PDACs.

The typical radiologic appearance of PNETs is manifested as a well-defined hypervascular solid nodule, with homogeneous and progressive enhancement, mostly in lower grade (20). The enhanced pattern can be used to predict tumor grade or help differentiate PNECs from PDACs (6, 15). Kim et al. (15) found that portal enhancement ratio less than 1.1 had achieved both high sensitivity and specificity (92.3% and 80.5%, respectively) in distinguishing G3 from G1/2, and G3 often presented with iso- or hypo-enhancement on portal venous phase at CT, which is in accordance with our study. Arterial/portal absolute enhancement emerged as independent factor for distinguishing between PNETs from PNECs exhibiting good diagnostic capacity indicating that the enhanced imaging pattern may reflect the tumor microenvironment and aid in the differentiation of these tumors (21). In our study, the portal enhancement ratio emerged as an independent variable for differentiating G3 PNETs and PDACs. Additionally, this index was found to predict the preoperative prognosis of PNETs with superior predictive performance than current staging systems (22).

PDACs are fatal diseases, with less than 20% of patients able to undergo surgery resulting in unsatisfactory outcomes. In contrast, PNETs are generally considered indolent lesions. However, G3 PNETs can exhibit an extra-aggressive nature and poor prognosis, especially PNECs (23). The median survival for patients with PNECs was 9.5 months, whereas well-differentiated PNETs could reach up to 43 months (8). The median survival for patients with locally advanced and metastatic PDACs ranges from 14 to 18 months (24).

A nomogram is a graphical tool widely used in clinical practice, based on multiple predictive factors, facilitating personalized risk assessment and treatment decision making (25, 26). A study identified tumor size, biliopancreatic duct dilation, lymph node enlargement, and enhancement pattern as independent factors for predicting the aggressiveness of non-functional PNETs, and a nomogram based on these features demonstrated good diagnostic performance (27). The nomogram could also function as a predictive tool for forecasting the likelihood of liver metastases or lymph node metastases in patients with PNETs (25, 28). Our nomogram contained age, pancreatic duct dilatation, and portal enhancement ratio to predict the probability of G3 PNETs. When the total score reaches 160 points, there is a likelihood of up to 90% for the presence of G3 PNETs. Conversely, if the total score is less than 90 points, the probability drops to less than 10%. Nomograms offer several advantages over traditional scoring systems, including improved accuracy, ease of use, and visual representation of risk factors (29).

This study possesses several limitations. First, the multicenter retrospective design led to the utilization of different scanners with varying parameters potentially resulting in unavoidable deviations. Second, there is a risk of introducing biases being a retrospective study. Third, we utilized consensus reading data as the primary means of image evaluation, without assessing interobserver agreement. Fourth, we did not differentiate PNECs from G3 PNETs despite their differing survival outcomes. Further analysis with a larger dataset is required. Last, owing to the relatively small sample size, we refrained from validating our results using additional cohorts. Further investigation with larger sample sizes and validation tests is warranted.

In conclusion, the nomogram model based on age, pancreatic duct dilatation, and portal enhancement ratio demonstrates good accuracy and discriminative ability effectively predicting the probability of G3 PNETs from PDACs. Furthermore, patients with G3 PNETs exhibit better prognosis than PDACs.

## Data Availability

The data analyzed in this study is subject to the following licenses/restrictions: Author could ask for corresponding author for the dataset. Requests to access these datasets should be directed to HD, 1182709239@qq.com.
